# The pattern of mucocutaneous disorders in HIV – infected children attending care and treatment centres in Dar es Salaam, Tanzania

**DOI:** 10.1186/1471-2458-9-234

**Published:** 2009-07-14

**Authors:** Millembe F Panya, Yassin M Mgonda, Augustine W Massawe

**Affiliations:** 1 Department of Paediatrics and Child Health, Muhimbili University of Health and Allied Sciences, Muhimbili, Dar es Salaam, Tanzania; 2 Department of Internal Medicine, Muhimbili University of Health and Allied Sciences, Muhimbili, Dar es Salaam, Tanzania

## Abstract

**Background:**

HIV/AIDS is associated with a wide range of mucocutaneous disorders some of which are useful in the clinical staging and prognosis of the syndrome. There is paucity of information regarding the prevalence and pattern of mucocutaneous disorders among HIV infected children attending paediatric Care and Treatment Centres (CTC) in Dar es Salaam. Objective To determine the prevalence and pattern of mucocutaneous disorders among HIV infected children attending public paediatric 'Care and Treatment Centres' in Dar es Salaam.

**Methods:**

This was a cross sectional descriptive study involving public paediatric 'Care and Treatment Centres' in Dar es Salaam. Clinical information was obtained using a questionnaire. Dermatological examination was carried out in daylight. Investigations were taken as appropriate. Data was analysed using the Statistical Package for Social Sciences (SPSS) program version 10.0. Chi-squared and Fisher's exact tests were utilized. A p-value of less than 0.05 was considered statistically significant.

**Results:**

Three hundred and forty seven HIV infected children (52% males) attending CTCs were recruited into the study. Mucocutaneous disorders were encountered in 85% of them. There was no gender difference in the prevalence of the infective mucocutaneous disorders but males had a higher prevalence of non-infective/inflammatory dermatoses (58%) than females (42%) (p = 0.02). Overall, mucocutaneous disorders (infective + non infective) were more prevalent in advanced stages of HIV disease. Children with advanced HIV disease had a significantly increased frequency of fungal and viral infections (43% and 25% respectively than those with less advanced disease; 24% and 13% respectively (p = 0.01). Seventy four percent of the HIV-infected children with mucocutaneous disorders were already on ART.

**Conclusion:**

Mucocutaneous disorders among HIV infected children attending Care and Treatment Centres are common and highly variable. Comprehensive management should also emphasize on the management of mucocutaneous disorders.

## Background

Mucocutaneous disorders are common in both HIV-infected and non-infected children. Human Immunodeficiency virus infection causes a severe cellular immunodeficiency, which results in a greater susceptibility to infectious, inflammatory and malignant conditions [1]. In early HIV infection, most of the mucocutaneous dermatoses are similar to those observed in the non infected children. With progressive immunosuppression, mucocutaneous eruptions, become more common and include patterns which are atypical, difficult to diagnose and resistant to treatment [2-4]. *Candida albicans *infection presenting as extensive oral thrush or recalcitrant monilial diaper dermatitis is the most common and often the first manifestation of paediatric HIV infection. Bacterial infections including severe forms of staphylococcal impetigo, ecthyma and furuncles are also common [3,4]. Extensive molluscum contagiosum, *Herpes simplex *infection and plane warts are also common with lesions which are more widely distributed and very difficult to treat [3,4].

Non infective/inflammatory dermatoses like seborrhoeic eczema and Kaposi's sarcoma, which are common in adults in whom they become more widespread and refractory to treatment as CD4+ T-cell count declines, are exceptional in children [4-6]. Pruritic Papular Eruption of HIV/AIDS (PPE) is common in both adults and children with marked depletion of CD4+ T-lymphocytes [5,7].

In this era of Anti – Retroviral-Therapy (ART), the immune reconstruction induced by ART would be expected to significantly reduce the prevalence of many dermatological diseases as a consequence of a profound suppression of viral replication with the accompanying increase in CD4+ T-cell count [1,7].

In Dar es Salaam, there is still paucity of information regarding the prevalence of mucocutaneous disorders among HIV infected children attending 'Care and Treatment Centres'. This study was conducted in order to address this lack of data; the main objective being to describe the prevalence and pattern of mucocutaneous disorders in HIV infected children attending these centres in Dar es Salaam.

## Methods

### Study design and Setting

This was a descriptive cross-sectional study conducted at the public Paediatric HIV Care and Treatment Centres in Dar es Salaam.

### Sampling technique

Study subjects were recruited using the 'sampling proportional to size' technique whereby, the number of children to be included from each CTC depended on the pre-determined number of children visiting that centre. A sampling fraction of 24% was used to determine the minimum number of children from each centre. All HIV infected children aged up to 17 years, whose parents/guardians gave informed consent, were enrolled consecutively until the required sample size was obtained.

### Clinical and laboratory work-up

All participants were interviewed to obtain demographic and clinical information and whether or not they were on ART. Dermatological examination was done in daylight. Diagnoses of most dermatoses were done clinically. Where necessary, appropriate laboratory tests like skin scrapings, pus swabs and skin biopsies were performed to confirm the diagnoses. Lymphocyte subsets were determined using a FACS calibur machine (Becton-Dickinson USA 2005).

### Clinical staging and immunological classification of HIV/AIDS

Clinical staging was done according to the WHO paediatric staging of HIV/AIDS [8] while immunologic classification was done according to the CDC immunologic classification for HIV infected infants and children [9,10].

### Data analysis

Statistical analysis was done using the Statistical Package for Social Sciences (SPSS) program version 10.0 [11]. Chi-squared test was used for categorical variables while Fisher's exact test was used when the number was less than 5. A p-value of less than 0.05 was considered statistically significant.

### Ethical Issues

Ethical clearance was obtained from the Research and Publications Committee of the Muhimbili University of Health and Allied Sciences.

## Results

### Age and sex distribution of HIV-infected children attending CTCs

A total of 347 HIV infected children were recruited into the study (Table 1). Of these, 180 (52%) were males. The age ranged from 6 months to 16 years (mean 7.8 years ± 3.8 SD).

**Table 1:** Distribution of HIV infected children attending HIV CTC by age and sex (n = 347)

Age group (years)	Males n (%)	Females n (%)	Total n (%)
0–5	61 (34)	44 (26)	105 (30)
6–10	77 (43)	78 (47)	155 (45)
11–15	36 (20)	39 (23)	75 (22)
16 -≥ 17	6 (3)	6 (4)	12 (3)

Total	180 (52)	167 (48)	347 (100)

Overall, 85% (294/347) had mucocutaneous disorders. Eighty seven percent of the males and 83% of the females had one or more types of mucocutaneous disorders (p = 0.29). Among children aged up to 5 years, 88% had mucocutaneous disorders while for those aged 6 years and above, the prevalence was 83% (p = 0.59).

### The pattern of mucocutaneous disorders among HIV infected children

A wide range of mucocutaneous disorders was encountered as shown in table 2. The infectious dermatoses (fungal, viral, bacterial and parasitic infections) accounted for the majority of the skin diseases (87%) while the inflammatory dermatoses (PPE, atopic dermatitis, seborrhoeic dermatitis and non specific dermatitis) accounted for 69%. Many children had multiple skin conditions and were therefore counted more than once in certain situations. When specific mucocutaneous disorders were analysed, PPE was the commonest condition present in 45.5% followed by superficial fungal infections in 40% of the children. The most frequent superficial fungal infection was tinea capitis, occurring in 16% while the least frequent was tinea cruris found in only one child. Viral and bacterial skin infections were found in 23% and 12% respectively. Plane warts found in 20%, were the most frequent viral infections while herpes zoster present in 2 patients was the least frequent. The commonest bacterial skin infection was bullous impetigo in 9.5%. The only neoplasm encountered was Kaposi's sarcoma present in one who had been on ART for 1 month. Two children had nail hyperpigmentation while one child had severe drug eruption (Stevens-Johnson syndrome) induced by co-trimoxazole.

**Table 2:** Mucocutaneous disorders in HIV infected children attending Care and Treatment Centres in Dar es Salaam (n= 294)

**Mucocutaneous disorder**	**N**	**%**
**Non infectious inflammatory dermatoses**	**202**	**69**
Pruritic papular eruption (PPE)	134	45.5
Atopic dermatitis	31	10.5
Seborrheic dermatitis	24	8
Non specific dermatitis	13	4.4
**Infectious dermatoses**	**256**	**87**
**Superficial fungal infections**	**118**	**40**
Tinea capitis	47	16
Tinea unguim	31	10.5
Tinea corporis	28	9.5
Pityriasis versicolor	11	4
Oral candidosis	11	4
Tinea cruris	1	0.3
**Viral infections**	**68**	**23**
Plane warts	58	20
Molluscun contagiosum	13	4
Herpes simplex	4	1.4
Herpes zoster	2	0.7
**Bacterial infections**	**36**	**12**
Impetigo	28	9.5
Abscess	6	2.0
Cellulitis	3	1.0
**Scabies**	**34**	**12**
**Kaposis Sarcoma**	**1**	**0.3**
**Drug reaction**	**1**	**0.3**
**Nail hyperpigmentation**	**1**	**0.3**

Table 3 shows the distribution of the different types of mucocutaneous disorders by age-groups. Fungal infections were more prevalent in the older children aged above 15 years (58%), while those aged 5 years and below were the least (20%) affected (p = 0.01). The prevalence of viral, bacterial, and inflammatory cutaneous disorders did not show any significant age-group difference (p ≥ 0.25).

**Table 3:** Distribution of types of mucocutaneous disorders among HIV infected children by age

MUCOCUTANEOUS DISORDER	AGE GROUP (years)				Total	p-value
	0–5 n = 105 (%)	6–10 n = 155 (%)	11–15 n = 75 (%)	>15 n = 12 (%)		
Inflammatory dermatoses	66 (63)	78 (50)	34 (45)	8 (67)	186	0.71
Fungal infections	21 (20)	60 (39)	30 (40)	7 (58)	118	0.01
Viral infections	15 (14)	28 (14)	22 (29)	3 (25)	68	0.35
Bacterial infection	10 (9.5)	17 (11)	9 (12)	0 (0.0)	36	0.93
Infestation (scabies)	14 (13)	11 (7)	7 (9)	2 (17)	34	0.25
Miscellaneous	1 (1)	0 (0)	2 (3)	0 (0)	3	0.76

### The pattern of mucocutaneous disorders by WHO and CDC level of immunosuppression

Table 4 shows the prevalence of mucocutaneous disorders by WHO clinical staging and CDC level of immunosuppression. The prevalence of mucocutaneous disorders was highest in the advanced paediatric WHO stages III (94%) and IV (100%) and relatively lower in stage II (76%) while it was lowest (29%) in stage I (p < 0.01). Likewise, children with severe immunosuppression by CDC criteria had the highest prevalence of mucocutaneous disorders (97%), followed by those with moderate immunosuppression (84.5%) while children with no evidence of immunosuppression had the lowest (71%) prevalence (p < 0.01).

**Table 4:** Prevalence of mucocutaneous disorders among HIV infected children by WHO and CDC disease progression (n = 347)

	Mucocutaneous disorders: **Present** n = 294 (%)	Mucocutaneous disorders: **Absent** n = 53 (%)	Total n = 347 (%)	p-value
**WHO paediatric stage**				
I	2 (29)	5 (71)	7 (2)	
II	116 (76)	37 (24)	153 (44)	<0.01
III	166 (94)	11 (6)	177 (51)	
IV	10 (100)	0 (0)	10 (3)	
**Immunosuppression level by CDC**				
No evidence	74 (71)	30 (29)	104 (30)	
Moderate	109 (84.5)	20 (15.5)	129 (37)	<0.01
Severe	111 (97)	3 (3)	114 (33)	

Of the 294 children with mucocutaneous disorders, about three- quarters (74%) were on ART according to the Tanzanian National HIV treatment guidelines. Of these, only 20% had been on ART for a period longer than 12 months. The majority (49%), had been on ART for a period between six and twelve months while one-third (31%) had used ART for less than 6 months.

## Discussion

From this study, 85% of HIV/AIDS paediatric patients attending CTCs in Dar es Salaam suffer from a wide range of mucocutaneous disorders. A high prevalence of skin diseases among HIV infected children similar to ours has also been reported by Montri et al in Thailand (83%) [12] and De Carvalho OV et al in Brazil (82.5%) [13]. Other studies have reported different prevalence levels [1,14] indicating a wide regional variation. According to Luminous LM et al, children less than five years of age, are the least affected than the older ones [15]. In this study, children aged less than 5 years were almost equally affected as those aged 6 years and above regardless of sex. Siriwan W et al reported the prevalence of mucocutaneous disorders in HIV infected children with severe, moderate and no evidence of immunosuppression as 62%, 43% and 20% respectively [14]. In this study, the prevalence of mucocutaneous disorders among children with severe, moderately severe and no evidence of immunosuppression was 97%, 84.5% and 71% respectively and the differences were statistically significant. All these studies indicate that the risk of acquiring mucocutaneous disorders for HIV infected children rises as the level of immunosuppression advances.

Infections are the most frequent cause of mucocutaneous disorders among HIV infected children [1,3,4,14] which has also been shown in this study. The increased incidence of skin infections is attributed to the depletion of the Langerhan's cells responsible for the mucocutaneous immunological system [13]. The distortion of the cutaneous immune system is also responsible for the emergence of a variety of non-infectious inflammatory dermatoses frequently encountered among HIV/AIDS individuals like PPE. In this study PPE was present in nearly half (45.5%) of the affected children.

In our study, Kaposi's sarcoma (KS) was seen in only one child indicating that this malignancy is rare among HIV infected children, which is similar to what was reported by el Hachem M et al [4]. However, other studies have reported a significant rise in childhood KS among HIV infected children. Ziegler et al reported that the incidence of KS had risen more than 40-fold in the era of HIV [16]. Similarly Athale OH et al observed an increased incidence of KS in HIV infected Zambian children [17]. It is surprising that, only one child was found to have a drug reaction (Stevens-Johnson syndrome). This finding probably does not reflect a true picture and calls for an appropriately designed follow-up rather than a cross-sectional study in order to assess the effects of all drugs administered at CTCs.

Nearly 75% of children with mucocutaneous disorders were already on ART according to the Tanzanian National Guidelines on Treatment of HIV/AIDS. The use of ART has shown a significant reduction in the prevalence of some of the mucocutaneous disorders in HIV infection as a consequence of an increase in CD4+ T cell lymphocytes [1]. It was not possible to demonstrate this effect in our study, because of the study design, which was cross-sectional rather than follow-up. The apparent high prevalence of mucocutaneous disorders among these children, who were already on ART at the time of this study, could be due to the short ART duration. The majority of these children (49%) were on ART for six to twelve months, while one-third had ART for less than six months and only about 20% had used it for more than one year. For ART to have a significant effect on mucocutaneous disorders, it needs to be administered for a longer period of time. In a study by Donic I. et al, it was found that the use of ART for about two years reduced significantly the presence of oral candidosis and seborrheic dermatitis [18].

## Conclusion

Mucocutaneous disorders among HIV infected children attending CTCs in Dar es Salaam, regardless of gender or age-group are common, highly variable and mostly infective. Their prevalence rises with deteriorating WHO clinical stage as well as CDC level of immunosuppression. Comprehensive management of HIV infected children attending CTCs should also emphasize on the management of mucocutaneous disorders.

## Study limitations

This was a cross-sectional study which could not offer an opportunity to assess the progress of the pre-existing mucocutaneous disorders, or document on the newly evolving lesions while children continued receiving care from the CTCs

## Competing interests

The authors declare that they have no competing interests.

## Authors' contributions

MFP conceived the study and participated in its design, data collection and write-up. YMM improved and supervised the conceived study, participated in its design, coordinated and approved the final write-up as corresponding author. AWM co-supervised the study and participated in its design. All authors read and approved the final manuscript.

## Pre-publication history

The pre-publication history for this paper can be accessed here:


